# Misinformation in Social Media Narratives on Highly Pathogenic Avian Influenza: Systematic Content Analysis of Facebook and Instagram Posts

**DOI:** 10.2196/73275

**Published:** 2026-04-09

**Authors:** Ahmed Al-Rawi, Betty Ackah, Abdelrahman Fakida, Kelley Lee

**Affiliations:** 1 School of Communication, Simon Fraser University, Schrum Science Centre-K 9653, Burnaby, BC, V5A1S6, Canada, 1 (778) 782-3117; 2 Faculty of Health Sciences, Simon Fraser University, Burnaby, BC, Canada

**Keywords:** highly pathogenic avian influenza, H5N1, social media, misinformation, health communication

## Abstract

**Background:**

Recurrent outbreaks of the highly pathogenic avian influenza (HPAI) A (H5N1) virus in farmed poultry, and reports of infections in dairy cattle herds in the United States since March 2024, have triggered concerns about the spillover threat to human populations and a subsequent influenza pandemic. The increasing threat that H5N1 poses to human health has led to more vigilant public health monitoring of these developments. In addition to intensifying surveillance, preventative strategies—like vaccinating those at higher risk—are being evaluated to help minimize infection and spread.

**Objective:**

Efforts to mitigate and respond to such an event will entail broad public health interventions including vaccination. However, analysis of the COVID-19 pandemic suggests that information quality can significantly impact the effectiveness of such measures by influencing public understanding and trust. Misinformation about H5N1 and other viruses circulating online often includes inaccurate information about transmission, prevention, and the severity of the viruses. By systematically analyzing these false narratives, public health authorities can better tailor their pandemic prevention, preparedness, and response strategies.

**Methods:**

In light of the emerging threat of H5N1, we analyzed the content of social media posts from Facebook (approximately 350,000) and Instagram (n=69,551) related to HPAI. Using 40 keywords associated with misinformation, we identified over 500 posts explicitly mentioning H5N1 and related terms for further systematic analysis. Posts were coded to identify targets and topics in the social media narratives. The “target” refers to the organization or person mentioned in the post, while the “topic” refers to the primary issue or subject being addressed.

**Results:**

Our content analysis identifies 7 main targets of misinformation, including government (149/544, 27%), health authorities (108/544, 20%), and international organizations (74/544, 14%). Also, from the 6 topics that have been identified, we found that the most widespread one was that authority figures purposefully engineer pandemics to achieve multiple political, economic, and other objectives (362/544, 67%) followed by societal destruction (121/544, 22%), and anti-vaccination (84/544, 15%). Other themes include societal destruction and religious allusions and prophecies.

**Conclusions:**

Our analysis of online content showed that H5N1 misinformation was primarily aimed at individuals or groups with differing degrees of political or institutional authority, such as government leaders and public health officials. These figures were often the focus due to their involvement in making health policy decisions and implementing public health measures. Decision-making entities and individuals were the target of various misinformation narratives. Results demonstrate the ongoing need for monitoring health misinformation to inform evolving public health responses to HPAI.

## Introduction

The highly pathogenic avian influenza (HPAI) H5N1 virus was first identified in poultry flocks in 1959, with the first human infection detected in 1997 [1-3]. The virus has since circulated worldwide, causing serious outbreaks in wild and domesticated birds, with periodic human cases reported [4]. Since 2021, H5N1 has been identified in a growing number of other wild, domestic, and zoo mammals (48 species by August 2024), in some cases causing major die-offs [5,6]. In March 2022, infections were first detected in dairy cattle in the United States, with more than 700 herds confirmed as infected by January 2025 [7]. Around 40 human infections linked to dairy cattle by January 2025 have raised concerns about the possibility of an influenza pandemic in human populations [8]. While the current assessed risk of human infection ranges in severity from low to medium symptoms compared to earlier assessments [4,9], the further spread of H5N1 to other mammals increases the potential for genetic reassortment that promotes further spillover events and sustained human-to-human transmission.

The growing risk to human health from H5N1 has prompted stronger public health monitoring to these unfolding events. Alongside heightened surveillance efforts, preventative measures such as vaccination of high-risk populations are being considered to reduce infection and transmission. However, substantial evidence suggests that the ability to implement such measures could be limited in the wake of the COVID-19 pandemic [10]. Public trust was a key factor in achieving effective public health outcomes during COVID-19 specifically to achieve public compliance with recommended measures [11]. Online misinformation has been found to erode public trust, which has, in turn, facilitated vaccine hesitancy and refusal [12]. However, levels of public trust in the United States in key health agencies declined between June 2023 and December 2024, with 53% (down from 64%) trusting the Food and Drug Administration to make the right recommendations. Sixty-one percent (down from 66%) trust the recommendations of the US Centers for Disease Control and Prevention (CDC) [13]. While there are multiple factors contributing to increased mistrust, a decline in quality of the health information environment is a key issue.

This study seeks to inform emerging public health responses to H5N1 by understanding the content of misinformation on selected social media platforms related to H5N1. To date, there has been limited study of this subject. In part, this is due to increasing controls put into place by social media companies regarding access to data including for scholarly purposes [14]. For instance, X (formerly Twitter) discontinued its free Academic API (application programming interface) in February 2023. Meta’s CrowdTangle, which allowed researchers free but limited access to data from Facebook and Instagram, is no longer available from August 14, 2024 [15,16]. This reduced access poses new challenges for understanding trends in misinformation. Using CrowdTangle and another data collection tool, this study navigates these barriers to understand the targets and topics of misinformation related to H5N1 on Facebook and Instagram. The findings show that such data can offer useful insights when monitoring social and behavioral factors that may impact public health efforts to mitigate or control a public health emergency [17].

Studies show that the ubiquity of internet connectivity in many societies has now made social media platforms primary information sources and means of communication [18]. Social media serve as virtual spaces for interactive engagements, particularly during times of uncertainty such as public health emergencies [19]. While these technologies enable connectivity in a globalized world, they can also be the source and rapid spreader of low-quality information [20]. This includes misinformation defined as misleading, inaccurate, or false information, although in some cases, there might be a facet of truth. Misinformation is distinguished from disinformation primarily by intent. Disinformation refers to the deliberate spread of false content with the intent to mislead or misinform [21,22]. However, due to the challenges of deducing the intent behind false information, we use the term misinformation to collectively describe all content in our dataset that is associated with falsehood or misleading claims. This approach is aligned with the trend by researchers toward understanding information quality separate from intent [23]. There is substantial evidence that both are part of what has been termed the post-truth world where people reject objective facts in favor of information aligned with ideology, identity, and other biases over verified facts [24,25]. This resulted in a proliferation of health misinformation across social media platforms during the COVID-19 pandemic. Moreover, this trend is likely to continue, and even worsen, as social media platforms such as X and Meta discard commitments to fact-checking in favor of leaving online communities “to decide when posts are potentially misleading and need more context” [26].

There remains limited but growing scholarly study on the content of misinformation related to H5N1. Studies show that misinformation concerning infectious diseases is more substantial and spreads more widely and quickly on social media than verifiable information [27-29]. Previous studies to date have analyzed the relationship between misinformation and a broad range of infectious diseases such as Ebola, H1N1, and Middle East Respiratory Syndrome [25,30,31]. Other studies have investigated social media misinformation against vaccination [27].

The structural features of social media platforms further the spread of misinformation as they often prioritize sensational content. Vosoughi et al [28] examined approximately 126,000 stories tweeted by about 3 million users on X to reveal that misinformation diffused at a significantly accelerated rate than verifiable information. Characteristics such as their novelty and emotional intensity facilitate their rapid diffusion. This phenomenon is particularly evident during health crises like avian influenza outbreaks [32].

For example, Yan et al [33] explore the relationship between social networking status, that is, the modes of information diffusion, relationship building, specific algorithmic exigencies, and influenza susceptibility. Gori and colleagues [34] examined social media debates on vaccines during the early part of the COVID-19 pandemic to understand the dynamics of vaccine readiness in Italy. The study highlighted how online debates using anti-vaccination rhetoric can influence the behavior of the vaccine-hesitant compared to pro-vaccination arguments.

Other studies posit the salience of factors such as people’s reliance on these platforms as primary sources of news and individual-level predispositions to a conspiratorial mindset [35,36]. As well, public concerns about the impacts of infectious disease outbreaks on social media have intensified in the wake of the COVID-19 pandemic. These underlying factors have amplified the rapid spread of misinformation, contributing to the rise of infodemics during epidemics and pandemics. They present significant dangers, leading to public panic, reducing trust in health authorities, and resulting in uninformed health decisions that inhibit optimal uptake of public health interventions. People who adopt inaccurate information about disease events are more likely to reject factual information about their severity, thus inhibiting their willingness to comply with appropriate measures [37].

Evidence suggests that the public’s interpretation of information on infectious diseases on social media platforms can influence behaviors in response to relevant interventions, which, in turn, can impact the trajectory of a health crisis [38]. This is particularly important for early interventions during an emerging crisis that have the potential to become pandemics [39]. Health crises tend to heighten levels of public uncertainty, anxiety, and fear. Studies show that online spaces offer opportunities for people to engage with social networks and information sources to allay their concerns [40,41]. However, as Wang et al [29] found, elevated levels of anxiety and fear can make people more susceptible to poor quality information.

The above research has identified best practices for health communication to attend to public concerns, facilitate appropriate interpretation of the information, and support trust in public health interventions [17]. For instance, the enduring impact of COVID-19 has affected people’s trust toward organizations like the US CDC and the government, in some cases augmenting an aversion to vaccination in general. Knowing these factors is key for appropriate communication framing [17]. Indeed, the COVID-19 pandemic showed how misinformation can significantly hamper public health interventions during health emergencies.

In this context, a better understanding of the content of misinformation on social media can inform effective public health responses to the evolving threat from H5N1 [42]. Misinformation on H5N1 may thus hinder public health efforts to identify, mitigate, or respond to the spread of the virus in animal and human populations by fostering further mistrust in public health responses [43]. Misinformation also has repercussions on individual and societal health outcomes by influencing health-seeking behaviors and compliance with public health interventions [17,29,44]. Although there are different subtypes of highly pathogenic avian influenza, such as H5N6 and H7N9 that have raised concern as well and remained important to analyze, our scope focuses on H5N1 due to its role as a primary subtype on known global outbreaks and designated by health authorities as a virus with pandemic potential. Moreover, H5N1 received wide media and public attention, making it particularly relevant for a study on health misinformation [45]. In sum, research on health misinformation related to H5N1 is limited, which this study seeks to address amid the ongoing spread of the virus. Thus, this study aims to answer the following research question: What is the content of misinformation about H5N1 on social media and how can this inform public health efforts at pandemic prevention, preparedness, and response?

## Methods

### Overview

Using scientific and colloquial terms associated with HPAI and specifically H5N1, such as bird flu, avian influenza, and avian flu, we collected approximately 350,000 Facebook and Instagram posts referencing H5N1 and associated terms using both CrowdTangle and a webometric tool called Zeeschuimer for more Instagram data. The data included both textual and multimedia content. We focused on these platforms because they are among the 3 most accessed social media platforms globally. Facebook is the market leader with an estimated active user population of over 3 billion [46]. Although YouTube is ahead of Instagram as the second most popular platform, its content being primarily video-based, with minimal text, excluded it from this study. We also selected Facebook and Instagram to expand the knowledge base on health misinformation beyond the platform X, especially regarding the affordances of social media platforms.

The CrowdTangle data were collected from June 5, 2014, to June 4, 2024, the posts are not limited to geographical demarcations. We collected the additional Instagram data with Zeeschuimer in July 2024. The total Instagram posts were (n=69,551). For both the Facebook and Instagram datasets, we employed a Python script to identify misinformation-related posts using terms such as “plandemic,” “medicalmurder,” and “LuciferShot” [47,48]. We compiled this list of terms based on research on health misinformation from academic literature, [49,50], online exploratory observations, and notes from the research team. Keywords were specifically focused on pandemic and vaccine conspiracy–related terms, demonstrating general connections for misinformation narratives between COVID-19 and vaccines. The study period from June 5, 2014, to June 5, 2024, was selected to ensure a proper review of discussions on the virus and its continued activity and outbreaks. A specific instance occurred in March 2024 when H5N1 infections were reported in dairy cattle in the United States for the first time, highlighting its continued relevance for public discussions [51].

Our final dataset contains 544 posts after removing duplicates. Though the volume of posts flagged as misinformation and misleading appears to be limited, we note several limitations. First, and in searching for specific keywords, it is difficult to confirm the identification of all the available misleading or misinformation-filled posts in the dataset, but the above approach has been effective in identifying a relevant subset of data that we can further explore. In addition, since the two data collection tools are not comprehensive in getting data from Meta, we cannot make any claims about the volume of misinformation on Facebook and Instagram. Moreover, as the initial number of gathered posts (350,000) is far larger than the remaining posts we analyzed, the difference in numbers is due to our inclusion criteria that excluded any duplicates, irrelevant use of keywords, and posts beyond our scope. This reduction also represents the inclusion criteria to ensure relevance.

Employing a general inductive approach [52] as part of systematic content analysis approach, 3 coders carried out a close reading of a 10% sample of the dataset to outline the emerging and dominant arguments. The analysis was multimodal, which involves the text and any associated multimedia content. Following a process of several rounds of phased discussions, the researchers organized the broad themes into 2 categories (targets and topics). These categories were used to link the analysis to the research objectives of understanding the prevailing misinformative concepts on HPAI to inform effective public health agenda. The target is the organization or individual discussed in the post. The topic concerns the main subject or issue in the post, ensuring that subcategories were mutually exclusive and that the themes of the various subcategories are unique to each category, while the central messages do not overlap. Two researchers then used this initial codebook on a sample of the first 100 posts to test the codebook and test intercoder reliability. After the first coding round, the researchers adjusted the target section of the codebook, adding corporations as a category to account for multinational and other commercial entities, along with a category for irrelevant posts. The codebook has nine categories under target: (1) government had the highest occurring target references (n=149), (2) public health authorities had 108 references, (3) international organizations (n=74), (4) elites (n=64), (5) pharmaceutical industries (n=52), (6) mainstream media (n=43), and (7) corporations (n=23). The final categories for targets were (8) other/unclear (n=113) and (9) irrelevant (n=53). We distinguished between these 2 categories because while some posts were focused on H5N1 but did not address a target (other), others did have targets but had topics that were unrelated to avian influenza (irrelevant).

Researchers also adjusted the topics section following the first round of coding to address vagueness and overlapping categories. After the second round of intercoder reliability, further adjustments were made, including renaming a category to religious allusions and prophecies to include a broader range of religious content. Moreover, we removed an alternative medicine category as there were only 2 posts, and they were better classified under other. The adjusted seven categories are (1) pandemic engineering, which accounted for 362 topics, (2) societal destruction (n=121, 22%), (3) anti-vaccination (n=84, 15%), (4) elections/democracy (n=66, 12%), (5) unjustified food insecurity (n=59, 11%), (6) religious allusions and prophecies (n=26, 5%), and (7) other/irrelevant (n=77, 14%). The final category contained posts that were not focused on H5N1 but on other pathogens and diseases such as SARS-CoV-2/COVID-19. Others did not have a clear message and sometimes also used contradictory hashtags [49]. There were also 2 posts advancing misinformative pseudoscientific remedies to H5N1 infections by advertising plant-based vaccines. After 3 attempts involving the modifications described above, intercoder reliability was statistically acceptable at 0.887 for targets and 0.899 for topics using Krippendorff Alpha [53]. Though limited contextual insight was identified due to lack of context, we also examined the nature of the social media posters by reviewing their profiles and previous posts. Since the evidence we collected on users’ profiles was only anecdotal, we did not report these findings in this study. Finally, as we analyzed publicly available social media data, all posts used have been deidentified and paraphrased to prevent re-identification and to protect user privacy.

### Ethical Considerations

This project received an exemption from the requirements of Research Ethics Review at Simon Fraser University (30002857). All analysis is based on publicly available and deidentified data. All publicly available social media content used in this study was deidentified to prevent reidentification of posts or individuals.

## Results

### Targets

#### Types of Targets

**Figure 1.**
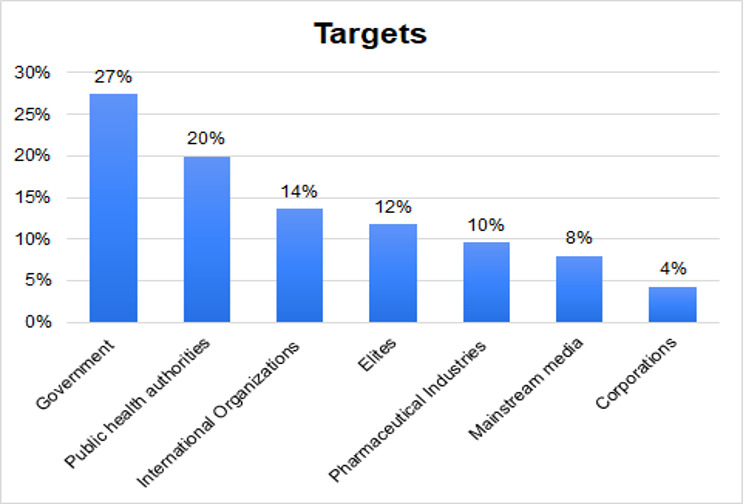
Percentage distribution of targets of misinformation on H5N1.

The identified targets (see Figure 1) were individuals or entities that possess varying levels of economic, political, or decision-making power. An underlying theme in the claims made against them centered on implying that they intentionally generated public concern.

#### Government

This category constituted 27% (149/544) of targets, with figures being rounded off to the nearest whole number. The posts addressed the roles and responsibilities of governments and governmental entities in relation to pandemics, including heads of states, ministries, officials, and agencies in different countries, and the United States Food and Drug Administration. The category included posts claiming that the government and its “deep state” actors created pandemics for their own benefit such as to influence elections or to benefit other corrupt actors and individuals. Posts often highlighted the COVID-19 pandemic measures that users claimed resulted in societal destruction, suggesting that warnings of avian influenza can lead governments to reimpose similar restrictions. The following paraphrased post illustrates this point:

*Don’t worry about #covid_19 the government is scaring you and lying to you to start a new digital money system It’s the same pattern they used before to [expletive] us and have reasons to push another PLANDEMIC. [expletive] them!*

In this example, wherein posts may contain typos as they are reproduced verbatim, the user points out different health crises such as COVID-19, Ebola, and avian influenza, suggesting that these crises were falsely created by the government as part of a larger conspiracy to impose certain financial measures on citizens. These are similar to other posts in the dataset in which users connect different health crises to argue that governments created or pushed these health crises to sow fear among citizens, thus creating conditions that allow the imposition of corrupt measures.

#### Public Health Authorities

Specific public health officials were targeted in posts, notably well-known figures such as Anthony Fauci, then director of the National Institute of Allergy and Infectious Diseases, and Robert Redfield, then director of the CDC. For instance, users frequently highlighted Redfield’s past warnings about the possible threat of an avian influenza pandemic as evidence of planned manipulation and creation of a “plandemic.” These posts also referred to public health officials as part of a broader network of parties that benefit from spreading fear about pandemics to advance individual interests. Public health officials comprised 20% (108/544) of the targets.

#### International Organizations

Fourteen percent (74/544) of the social media posts identified linked international organizations, especially those involved in pandemic responses such as the World Health Organization (WHO) and the World Economic Forum, with efforts to push political agendas that the posts claimed serve or align with their interests. This post, for example, accused the WHO of spreading false information regarding an avian influenza death to create a health crisis that can help control the public and push for certain medical interventions. The paraphrased excerpt below partly explains this claim:

*The terrorist global health care organization is making up stories about someone dying after being infected with #avian influenza… because those in power want to turn us into mindless people… to push their medicines and spread panic and keep us under their control. Don’t trust them; they are manipulating you. Make sure to focus on your healthy habits and keep learning and praying.*

#### Elites

Elites are individuals perceived by users to hold power and influence in the health sector and pandemic response such as Bill Gates, American businessman and philanthropist, and Klaus Schwab, the founder of the World Economic Forum. Constituting 12% (64/544) of the targets, the posts connected them with larger conspiracy theories, suggesting they had ambitions to reduce meat consumption, which was linked to perceived food shortages, and advanced the “Great Reset,” a theory claiming that the COVID-19 pandemic measures were orchestrated by global elites allegedly to achieve their own goals, such as the following paraphrased quote:

*It seems that the next Plandemic could be happening soon. This could be airborne and will be blamed on the new bird flu strain. Be careful, there is always another plan in the works. Billionaires already told us that they will go for our attention during the new pandemic, and the global health organizations and those globalists planned this new disease and created a Global Pandemic plan because they want to control our nations through a “Health” emergency.*

#### Pharmaceutical Industries

Posts focused on pharmaceutical companies accounted for 9.57% (52/544) of posts. Several users who promoted skeptical messages about avian influenza and pandemics highlighted the role they claimed these pharmaceutical companies played with other discussed entities such as the government and international organizations to amplify perceived medical threats and eventually sell the vaccines these companies produced. For instance, one post commented on avian influenza-related news saying that “psychic vaccine makers” prepared a vaccine for avian influenza a year in advance, suggesting that this is a “plandemic.” Such posts reference vaccine developments as evidence that pandemics are intentionally created to help vaccine manufacturers.

#### Mainstream Media

Posts targeting mainstream media amounted to approximately 8% (43/544). Users who criticized mainstream media outlets portrayed them as tools used to spread fear about pandemics and diseases, and sometimes to encourage people to take vaccines, by claiming that they are manipulating and exaggerating to eventually support corruption that allows governments and pharmaceutical companies to benefit from pandemics.

#### Other Private Companies

Four percent (23/544) of the posts identified targeted nonpharmaceutical companies. Among the allegations were claims that various companies influence governments and organizations to declare pandemics, given that these companies were described as profiting from health crises. Some posts alleged that food companies, for example, pushed governments to use avian influenza to reduce the number of poultry in small farms or to mandate vaccines that could harm chickens. Such claims argue that these corrupt measures create food shortages that eventually corporations that control poultry supply will benefit from.

### Topics

#### Types of Topics

**Figure 2.**
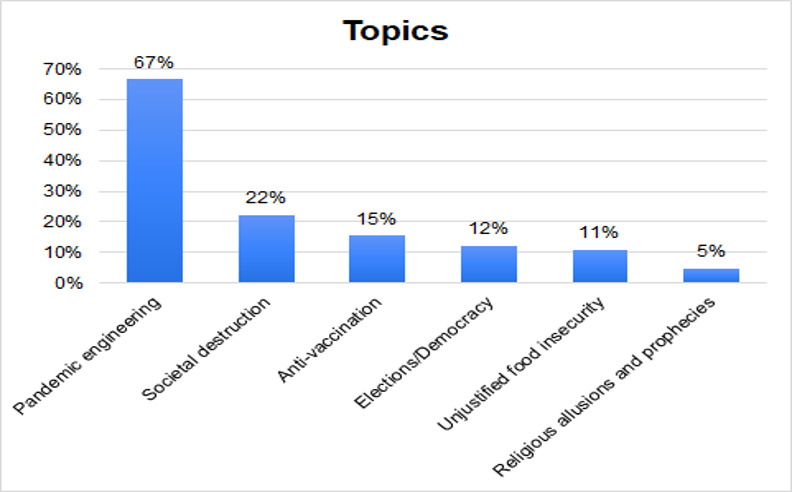
Percentage distribution of H5N1 misinformation topics.

The dataset included an extensive range of topics that were categorized according to the most prevalent narrative arguments (see Figure 2). The proportion of posts referring to the engineering of infectious disease pandemics was significant. The topics largely attributed major infectious disease events to human actions rather than any roles that pathogens play.

#### Pandemic Engineering

These posts (362/544, 67%) claim that the H5N1 outbreaks were allegedly engineered. They claimed that viruses causing widespread outbreaks are fabricated bioweapons aimed at stimulating economic gain for their creators or enshrining political power for governments or international organizations. Other targets of pandemic engineering claims are pharmaceutical companies and elites like Bill Gates. Popular hashtags associated with this theme include “#scamdemic” and “#plandemic.” This post illustrates narratives about antiviral medications being specifically designed as bioweapons to manipulate people’s beliefs such as the following paraphrased claim:

*That is strange, they are already creating a new medication for the new flu. This drug and its company already know all the potential side effects …including sensory symptoms and continued false beliefs. The company that created the drug could just be saying that side effects could seem like a zombie apocalypse…They are clearly experimenting these medications on us to change our thoughts and memories!*

#### Societal Destruction

Amounting to 22% (121/544) of posts, this category is made up of posts that described widespread negative consequences from H5N1 outbreaks and public health measures. The consequences outlined were either projections of future occurrences or outcomes that are described as already happening, such as suicides and economic crashes. In the example below, the user indicated that pandemics of any kind could result in economic crashes and wars. Thus, alerts of avian influenza and swine flu infections were particularly disturbing in the wake of the COVID-19 pandemic and its ensuing effect. The paraphrased quote below further explains this point:

*Oh, no! Now there is pig flu in addition to covid? They are saying that influenza this year, will be bad. Pandemics can start economic disasters and economic issues can lead to conflict. The elites and globalists and others are creating a perfect storm.” It’s the same people who created the New World Order and are now using China to bring that “perfect storm” on Chinese people and affecting all of us.*

This post encapsulates multiple theories that proponents of misinformation disseminate. One is the idea that pandemics are created to intentionally destabilize the political and economic systems of the world. Amid the ensuing chaos, global hegemonic figures are portrayed as aiming to establish a new hierarchy, a New World Order. The transformed global landscape is generally framed in economic terms; dominant forces will gain tremendous wealth and concretize their power. In this instance, the misinformation posts deviate from systematic examinations of pandemic impacts by overwhelmingly focusing on the negative impacts of pandemics and a purported new world order, while rejecting the public health measures (such as vaccinations), which could help mitigate disease spread and therefore minimize the impact.

#### Anti-Vaccination

Anti-vaccination sentiments were the third most dominant topic at 15% (84/544). The example below demonstrates the majority of the reasons that users gave for rejecting vaccination, including the profiteering motivations driving pharmaceutical companies to manufacture vaccines and vaccines being allegedly used to implement a depopulation agenda. Others asserted that the vaccine manufacturing process is largely experimental and that not enough tests are run before they are administered to humans. Another conspiracy theory was that vaccines are used to insert digital biometric identity mechanisms into people, which would then be used to enforce a digital economy.

*This is not good! They will do anything for money. These companies and health care providers will specially target poor neighborhoods. Vaccines are hurting our children, they’re toxic and made of chicken embryos and from bird flu, pigeon flu, avian influenza, H1N1 and other viruses. Stop using our kids for testing drugs! Wake up and stop it!*

A notable aspect of misinformative posts against vaccines was how proponents seek to legitimize their messages by adapting “seeds” of facts. For instance, some posts refer to the history of cell strains originally derived from human fetal tissue in the development of some vaccines (from text in an image): “What’s in your needle?… measles virus…monkey kidneys…aborted fetal tissues…chicken embryo.”

#### Elections/Democracy

Posts discussing the connection between H5N1 and topics related to elections or democracy made up 12% (66/544) of the subset. Some users emphasized the political ramifications of an avian influenza outbreak. They include a perceived attempt by liberal governments to use outbreaks to curtail people’s rights and freedoms and a socialist takeover by China supported by the United States and other governments. These allegations were frequently presented in tandem with other conspiracy theories. Many posts thus contended that the attacks on democratic freedoms would be facilitated by the modified vaccines being used to digitally tag everyone. The perceived attacks on rights and freedoms are usually linked to people’s rejection of social distancing, vaccination, and other mandates, which were instituted in many countries during the COVID-19 outbreak. Posts frequently included multiple conspiracy-related narratives connected to reinforce the claim. In some cases, even contradictory assertions are believed to be true at the same time. This example from our dataset revealed the convergence of an engineered pandemic, fake climate change, and attempts to sabotage elections, all of which are being allegedly implemented by globalists.

*I’m actually worried about those who turned against their neighbors during the pandemic (while those who doubted all this were actually right); this is similar to how people snitched on their neighbors during wars. The same could happen when the next fake pandemic is created bird influenza? Climate change? The next political scheme and elections?*

#### Misinformation About the Food System

Among the actions that health authorities in Canada and internationally implemented to control H5N1 infections in farmed flocks are quarantines, restrictions on imports and exports, culling of birds, and disinfection of objects and premises. Another concern that was expressed in the dataset involved food security in relation to HPAI interventions such as depopulation of poultry farms. The posts below illustrate assertions about authorities’ intentional use of avian influenza to cause food insecurity by creating insufficiency in meat, eggs, and other food supplies.

*They are using fake results in PCR tests to kill animals and prepare for an Avian influenza. Plandemic and land misuse reduce the amount of food available…this is all to create a crisis and push people towards digital IDs and force them to take vaccinations and what’s worst is that they will replace meat with fake products and insects. We have to fight; this doesn’t support the corrupt system.*

The example demonstrates the above-discussed confluence of conspiracy theories, where proclamations of fabricated food crises feed into fears about digital identification, fossil fuels, and a new economic world order. Another post linking avian influenza to an agenda to advance insect and lab-grown meat diets included the following hashtags touting multiple theories since hashtags produce hypertexts [54]: #foodshortage #thegreatreset #depopulationagenda #newworldorderagenda #globalists #elites #climatechangehoax #clotshot #birdflu #populationcontrol #labgrownmeat #waronmeat #warmongers #billgatesisevil.

A final noteworthy theme under the food system is allegations that the Chinese government is gaining control over food supplies by unleashing H5N1 as a weapon of dominance. Misinformation on the food system was 11% (59/544) of the subset.

#### Religious Allusions and Prophecies

This category comprised approximately 5% (26/544). They all espoused Christian or Biblical allusions, including repentance from sin as an infection prevention method, apocalyptic prophecies on various catastrophes, which human societies will experience as the world nears the end of its lifespan, and vaccines being used to implant the mark of the beast. Chapter 13 of the Biblical Book of Revelation notes that during Earth’s final days, a beast will assume global leadership and require everyone to receive an identifying mark that will enable them to engage in societal transactions. The marks will be placed either on foreheads or on right hands.

*The prediction is about to happen; don’t accept these tech implants that they want to put in your hand and head. They want to add online money to your body. This bird flu is part of the next fake pandemic to spread digital identification and currency.*

The above illustrates misinformative claims that vaccines contained materials such as nanochips that marked immunized people with the mark of the beast. A recurring aspect of the messages in this category was the correlations made between announcements forecasting disease events and Biblical prophecies. Over time, and especially with reports of infections in mammals such as cows, various health experts have sounded warnings about imminent avian flu pandemics. News stories on such statements have bolstered religious myths, with people claiming that they were fulfillments of prophetic proclamations as shown in the example below.

*Remember that former public health official and what he said in this video. Remember when he mentioned that the next likely pandemic could be coming from bird flu?! He didn’t confirm that it will be for sure, but let’s keep an eye on this. We might see a biblical prophecy about wars and famines; these are the signs of the end times*.

[#Matthew 24:7 KJV].

## Discussion

### Principal Findings

The findings from this analysis of misinformation on 2 major social media platforms raise implications for public health policy and practice on the evolving H5N1 situation. Some evidence suggests that efforts to counter misinformation, such as debunking or fact-checking, may unintentionally amplify misinformation by elevating its perceived significance [32]. As the sample of posts analyzed represents a small portion of the initial dataset, our study does not aim to show prevalence on various platforms but to describe misinformation narratives and their types. The qualitative analysis of these narratives can demonstrate the construction and development of such narratives on this topic.

Importantly, findings suggest the need for the public health community to carefully consider such misinformation. Evidence shows that misinformation on social media has a differential diffusion pattern compared to traditional media [23]. Moreover, studies show that misinformation garners more engagement than facts. As Vosoughi et al [28] found, “falsehood diffused significantly farther, faster, deeper, and more broadly than the truth in all categories of information.” The top 1% of the most popular false news was disseminated among 1000 to 100,000 people. Verifiable information, however, barely reached more than 1000 people. Misinformation had a 70% higher chance of being retweeted compared to facts. By contrast, the truth took about 6 times as long as false information to reach 1500 people. This more rapid and far-reaching diffusion pattern of misinformation is also reported by studies of medical large language models, which have been found to be vulnerable to “data poisoning” attacks [55]. Large language models, trained on web-scraped data, are increasingly being explored to support health care [56]. However, as Alber et al [47] wrote, “replacement of just 0.001% of training tokens with medical misinformation results in harmful models more likely to propagate medical errors”. Thus, a deeper understanding is needed over time of their diffusion pattern and potential role in the spread of misinformation.

Our findings provide a baseline from which ongoing monitoring for trends in patterns of spread can be undertaken and lay the foundation for future work exploring misinformation on the HPAI virus in online spaces. For example, findings in this study about the use of scientific terminology in anti-vaccination posts showed similar points to the investigation by Faasse et al [57] of the dynamics of language use in pro- and anti-vaccination commentary on Facebook. They found that messages against vaccination used linguistic markers that resembled scientific language. Under the elections/democracy topic, posts analyzed reflected patterns noted in other studies regarding the belief in multiple conspiracy theories at the same time [36]. For instance, respondents in Miller’s study asserted that COVID-19 was a bioweapon manufactured by China, and the United States accidentally released it into society. In the study by Hellsten et al [58] on X communication networks during avian influenza outbreaks in the Netherlands from 2015 to 2017, they found frequent instances of concurring themes such as the rejection of health regulations #kippendans (chicken dance), #vuurwerk (firework), and #jacht (hunting). Thus, a principal recommendation is that public health systems need to use early warning data of social and behavioral factors, including social media monitoring of misinformation, to inform the development of public health messaging.

Second, our systematic analysis of the content of the posts deemed misinformation suggests that claims about H5N1 are an extension of dominant narratives, by topics and targets, put forth during the COVID-19 pandemic and other issue areas such as climate change. This could be seen, for example, in patterns of blaming a foreign entity or minority communities, such as blaming the Chinese government for H5N1, for a major disease outbreak, which has occurred regularly in history. Examples found included the massacre of Jews during the Black Death of the 15th century and during The Great Plague and the Spanish Flu of 1918‐1919; the lesbian, gay, bisexual, transgender/transsexual, queer, and other minority sexual orientations and gender identities community during the global AIDS pandemic; and Asian people during the COVID-19 pandemic [59].

An overarching theme as well was mistrust of the motivations for public health messaging and interventions. Our findings suggest that views sustaining mistrust persist, framed around a broader narrative of control by powerful elites to achieve a new world order favorable to their interests [60]. H5N1 has become subsumed within this world view, with public health concerns about the virus attributed to self-interested “pandemic engineering.” [61] Misinformation about the food system also demonstrated patterns of such mistrust in authorities following measures to control H5N1 infections such as bird depopulation. Bird depopulation can appear drastic because it involves both healthy and infected birds within a given perimeter. It has, however, shown to be the most effective method for stemming infection spread [8,62,63]. Misinformation against this measure typically has ominous labels such as murder and mass slaughtering. Additionally, while health authorities and governments have announced that H5N1 does not currently present food safety risks if animal products are appropriately processed or cooked [55,64], some misinformative posts called the safety of dairy products into question.

Another pattern of mistrust found was under religious allusions and prophecies topics, as users connected prominent public health communications, such as one made by the WHO Director-General [65], with religious narratives seen as the realization of prophetic predictions. This topic, in general, offers insights among certain religious groups that may resist mainstream information. Christian beliefs and those of other religious and insular communities are primarily grounded in faith rather than empirical evidence [66]. Such groups, particularly the fundamentalist ones, reject mainstream and external information, reinforcing their worldviews and possibly a greater risk of accepting misinformation [67-69]. Therefore, the credibility of such religious declarations relies on people’s acceptance of the parallels with Bible verses, which could reduce the need for expositions based on objective reality. Douglas further contends that the growing relationship between misinformation and religion is due in part to religious communities being principal targets [70]. This relates to the historical foundations of misinformation that was facilitated by Christian fundamentalists seeking alternative news sources as they rejected mainstream media’s inclination toward what they deem as biased expert knowledge, which usually deviates from religious dogma [6].

Given this broader framing, rebuilding and protecting public trust in the state, market, and civil society may require efforts beyond the public health community. Instead, strategies to sustain public trust in health practitioners and scientists, which remains high in most countries [71], as sources of quality information can be supported. For example, “localizing” public health efforts by working in meaningful partnership with community leaders can “humanize” and thus increase trust in public health messaging [72].

Third, our findings demonstrate the importance of protecting information quality as the H5N1 situation evolves while balancing the need to not draw undue attention to misinformation. Unlike COVID-19, the public health community has been managing the risks from H5N1 since the late 1990s, so much is already known about the virus [73]. Since 2022, the spread to a growing number of mammal species including dairy farms has increased media attention and public awareness [2]. Evidence suggests that there is thus an opportunity for public health messaging to facilitate public understanding of this issue while it is still emerging. For example, Vos and Buckner [74] found that sensemaking plays a key role in the success of public health interventions during health crises. Sensemaking involves the way the public interprets and frames health information they receive about emerging health events. The misinformation identified in this paper suggests H5N1 is already interpreted using existing conspiracy-related worldviews (eg, New World Order). To counter misinformed sensemaking, there is a need to avoid information vacuums. Moreover, public health messaging must not only be factually accurate but in a form that facilitates sensemaking. Accordingly, there is a need to provide appropriate information for the public to understand, and not just be informed, about H5N1. The insights into online health misinformation on HPAI that this study provides are instrumental for supplementing surveillance structures, guiding communication mechanisms with an understanding of the misgivings by some, which could be fueling hesitation toward and rejection of interventions and ultimately bolstering pandemic readiness measures.

Importantly, the ability to monitor the content and volume of misinformation on social media, and proactively message about the evolving H5N1 situation, is likely to be more important yet more challenging amid changing practices by social media platforms to limit access to data and to protect information quality. One of the strengths of this study is the novel expansion of online health misinformation analysis beyond the textual content of X platform. Our multimodal analysis of textual and multimedia data from Instagram and Facebook broadens the scope of knowledge of online public narratives, particularly about infectious diseases. Despite Meta’s claims about the strict moderation of posts,^75^ the majority of posts identified as misinformation were not flagged by both Facebook and Instagram for fact-checking. There were only 5 posts in our dataset that were flagged as inaccurate (less than 1%). The generic notification stated they were not completely factual, and they contained information similar to other posts that third-party fact checkers had reviewed. In 2018, CEO Mark Zuckerberg stated that Meta’s research showed, “that no matter where we draw the lines for what is allowed, as a piece of content gets close to that line (demarcating prohibited content which contains misinformation and harmful material), people will engage with it more on average—even when they tell us afterwards they don’t like the content” [76]. The announcement in January 2025 that Meta would end third-party fact-checking, in favor of allowing people to “express themselves freely,” could have implications for protecting information quality [77]. Given the ubiquity of social media and its centrality in sustaining social connection, especially during times of uncertainty such as public health emergencies, the public health community can benefit from understanding and navigating this evolving landscape as a core component of future pandemic preparedness and response.

### Limitations

A key limitation was the inability to include an analysis of the volume of misinformation on Meta platforms due to the lack of data collection tools. Moreover, we were unable to analyze the ideological leanings of users to further contextualize the narratives. We conducted a brief review of the profiles of users in our dataset to explore their potential ideological or political affiliations. Though some users’ profiles suggested specific political ideologies, this analysis was not included in the study, as confirming such affiliations would require an ethnographic approach, which falls outside the scope of this paper.

Additionally, our paper was limited to English-Language and region-specific search terms that mostly covered North America, along with a focus on some US public figures such as Anthony Fauci. This could have excluded similar discussions from other regions and countries such as the United Kingdom and Germany, where different terms were used. Future research could address this by including additional relevant keywords from different regions such as “flockdown” and “raw milk.” Another limitation was the prioritization of pandemic and vaccine-related terms in our dataset, which meant that other misinformation narratives about transmission and treatment, for example, may not have been captured. This limitation should be considered when examining our findings, as the topic categories identified were a result of our keyword search focus. Thus, findings reflect a specific focus of H5N1 misinformation.

### Conclusions

Misinformation on HPAI on social media has implications for public health as recurrent avian influenza outbreaks in animal populations have contributed to fears about the pandemic potential of the disease for humans. Notably, H5N1 in human populations is associated with several clinical symptoms; thus, decisive and swift actions are needed to stem any further infections. This finding underscores the importance of reviewing contextual factors like online misinformation, which could interfere with health interventions.

Our online investigation revealed that the targets of H5N1 misinformation narratives were largely people or entities who wield varying levels of political or other authority such as government and public health officials. These individuals received attention for their varying roles in health-related decision-making and in facilitating public health interventions.

We also found that the most prevalent misinformation themes were accusations of pandemic engineering, societal destruction, anti-vaccination sentiments, impact on elections or democracy, causing food insecurity, and religious allusions and prophecies. These assertions took various forms, including accusations that the virus itself did not occur naturally but claimed that the virus was artificially created as a bioweapon. The messages that contained anti-vaccination sentiments concluded that health interventions for avian influenza would include vaccines that would insert digital biometric identification into people, which could be used to either instigate population control or enforce a digital economy.

## Abbreviations

CDC: Centers for Disease Control and Prevention
HPAI: highly pathogenic avian influenza
WHO: World Health Organization
